# Application Optimization of University Aesthetic Education Resources Based on Few-Shot Learning from the Perspective of Ecological Aesthetic Education

**DOI:** 10.1155/2022/4248778

**Published:** 2022-04-22

**Authors:** Yang Liu

**Affiliations:** Hubei University of Education, Wuhan, Hubei 430205, China

## Abstract

The idea of EAE (Ecological Aesthetic Education) is put forward on the basis of the mature development of ecological aesthetics and AE theory. Starting with EAE, establishing people's aesthetic attitude and improving people's spiritual realm will help to reverse people's hostile attitude towards nature and rebuild the harmonious relationship between man and nature. This paper studies the application optimization of AE (aesthetic education) resources in universities based on ML (machine learning) from the perspective of resource development. The recommendation algorithm based on ML is the main idea of the classification of AE resources, and the classification model of AE resources is constructed. Through deep learning, we can learn the effective features of items from the content data in advance and then transform the learned features into CF (Collaborative Filtering) target learning task. Optimize the voting mechanism of the algorithm, and compare the RF_VM (random forest with optimized voting mechanism) algorithm with the traditional RF (random forest) algorithm. Experiments show that the algorithm proposed in this paper can effectively classify texts and has high feasibility.

## 1. Introduction

The core of basic education reform is curriculum reform, and AE is an aspect that cannot be ignored in curriculum reform. The development and utilization of AE curriculum resources is an important part of AE. The essence of EAE (Ecological Aesthetic Education) is AE with vivid ecological beauty, which exerts a subtle influence on college students, which is unmatched by other ecological education and AE forms [1]. It should be emphasized that the implementation of EAE is mainly realized through art education. That is to say, art education in our school must also examine its own value and function from the perspective of EAE. The effective implementation of EAE will help young people enjoy the fun of life no matter what stage they are in, and they will not give up easily no matter what adversity they face in the future [2]. School living resources are the foundation and premise of school EAE, and the development and utilization level of school EAE resources determines the effectiveness of school EAE.

The smooth implementation of education requires a well-designed school curriculum. Process is a means and tool for achieving educational and teaching objectives, as well as an important link in determining educational quality [3]. AE (Aesthetic education) is frequently based on theoretical research or individuals in terms of a comprehensive and systematic analysis of history, current situation, tasks, and in these papers [4–7], but there is still a lack of research on how to systematically and comprehensively apply AE elements. The content and quality inspection of the scientific AE process is the key to the development of college AE [8–10], while rationality is the issue that college AE does not play its role. This is a crucial reason, and more in-depth and systematic research and discussion on the use of AE resources in universities is required.

Generally speaking, the university AE started later than other education, so we have some knowledge of university AE, but there is still a big gap compared with other education. Although some university leaders attach importance to AE and care about building AE, not all university leaders attach importance to it and there is no general consensus. EAE development aids in the cultivation of people's ecological awareness, the realization of harmonious coexistence between man and nature, the improvement of people's spiritual world, and the realization of people's poetic existence. The active development of EAE is a key component of current AE. The goal of this study is to more objectively analyze and evaluate the selection, organization, and implementation of university AE resource support by looking into the current status of AE resources in universities and identifying problems with the support. Recognize the current state of AE resources in universities and propose countermeasures and suggestions that are appropriate for the goal.

Innovation:

1. Taking resource development as a breakthrough, this study makes theoretical thinking on the main topic of youth education-school EAE. In the study of school EAE, we should focus on resource development, explore the elements of EAE resources, choose an effective implementation path of school EAE, and develop and construct AE resources ecologically.
2. Personalized recommendation is very common on commercial websites, but it is rarely used in university AE resources. In this paper, the similarity of course and user attributes is calculated, specific algorithms are designed, and personalized recommendations are made so as to put forward reasonable suggestions for the application optimization of AE resources in universities.

The organizational structure of the paper is as follows:

The first chapter introduces the research background and significance before moving on to the paper's main work. The second chapter focuses on the technologies associated with university AE resource application optimization. The research's specific methods and implementation are presented in the third chapter. The fourth chapter verifies the research model's superiority and feasibility. The fifth chapter is a synopsis and preview of the entire text.

## 2. Related Work

### 2.1. Present Situation of AE Research in Universities

The fundamental goal of higher education is to promote the high-level development of college students, and the ultimate goal is the all-round development of morality, intelligence, physique, beauty, and labor. Among them, AE is not only one of the important goals of quality education, but also one of the important contents of school education. With the continuous promotion of quality education strategy in China, more and more scholars begin to pay attention to and study the AE in universities.

Aesthetic course research must be grounded in AE theory research. All students, regardless of their background or talent, should have the resources and conditions to learn art and exercise their right to art education, according to Leanne and others [11]. According to AFBY and others, professional art education in universities has advanced to unprecedented levels in the new era, with universities establishing art departments, offering art courses, organizing art activities in schools, and promoting a positive artistic environment in schools [12]. Many universities have not included AE in their education plans, but AE still exists, according to Jianlin et al. and this has had an impact on the development of AE practice [13]. Because of its own characteristics, Lvdn and others believe that AE, as one of the methods for achieving quality education, has an inherent and inevitable connection with moral education, intellectual education, and physical education and pervades all education, games, and people's spiritual worlds, and plays a significant role in comprehensive influence. Freedom, communication, experience, honesty, and extensiveness, according to Krainer and others, are the operating principles of modern AE. Yan and colleagues looked at teaching aesthetics from three perspectives: language, posture, and rhythm.

Although the research on the basic theory of AE has achieved fruitful results, the existing research on AE in universities has little penetration into the new achievements of AE theory and lacks the support for college students' AE theory.

### 2.2. Research Status of ML

ML (machine learning) [14–17] is the core of artificial intelligence and the fundamental way to make computers intelligent. Using effective artificial intelligence technology to obtain abstract information from big data and turn it into useful knowledge is one of the main challenges facing big data analysis today [18]. In the era of big data, how to effectively analyze complex data, reflect its value, and make rational use of it is an urgent task to be solved and considered.

Stein et al. conducted a study to eliminate heterogeneous influence by removing sensitive attributes from the decision-making process and adding fairness constraints to avoid heterogeneous processing. Observe that the standard fairness constraint is nonconvex; then use covariance to turn the nonconvex problem into a convex problem. Finally, measure the output results and sensitive attributes of parameters to investigate the sensitive attributes of multiple classification and analysis. Some scholars looked at the fairness metrics of a variety of algorithms to see how they correlated with different definitions and discovered that they were very closely related. The transformed data retains most of the characteristic signals of insensitive attributes, intersection-sensitive attributes are proposed, and the effects of two sensitive attributes do not overlap [19]. Nguyen et al. modified each attribute so that the marginal distribution of a given subset of sensitive attributes is the same, and this change will not affect other variables, and the transformed data retains most of the characteristic signals of insensitive attributes and intersection-sensitive attributes are proposed, and the effects of two sensitive attributes do. To control identification, limit the degree of distortion of a single data sample, and maintain utility, Nguyen et al. proposed a convex optimization for learning data conversion [20]. Nguyen et al. improved the logistic regression and support vector machine algorithms without bias based on historical data and provided a flexible balance between fairness and accuracy according to various false alarm rates. When sensitive attribute information is unavailable, this method works well [21]. In social networks, Wang et al. proposed a random walk push model. In the trust relationship, this model uses the traditional CF (Collaborative Filtering) method, which can define and measure the reliability of recommendations [22]. Lang et al. experimented with a number of online recommendation systems [23] and found that when both online systems and friends recommended them, users preferred the latter. The difference between content-based and user-based recommendations demonstrates that users prefer the latter. However, their model's goal is to predict users' and items' scores rather than personalized preference ranking, so the goal does not align with the recommendation system's ultimate goal, resulting in low recommendation accuracy.

## 3. Methodology

### 3.1. Analysis of the Application of AE Resources in Universities

Ecological problems are closely related to people, and ecological imbalance is the deviation of people's ideas and consciousness. To solve ecological problems, we must first solve the problem of people's ideology, which is actually a problem of people's education. AE aims to establish people's aesthetic attitude and create aesthetic living conditions, as an education to improve people's living standards. Starting with AE, it is of great significance to establish people's aesthetic attitude for solving ecological problems and improving people's living conditions, and EAE is still a new field of AE in China.

EAE's mission is to educate and infect people through art and various forms of EAE so that they can achieve true enlightenment, good education, and emotional edification in a pleasant spirit and gradually develop people's ecological aesthetic sensibility and consciousness. Realize the survival of ecology and aesthetics and perform a one-of-a-kind and irreplaceable role. On the one hand, we should focus on infecting and educating people about the aesthetic method of perceptual freedom so that they can consciously educate beauty or “give freedom with freedom.” On the other hand, understanding the natural laws of the ecosystem should be a priority in the implementation of EAE so that people can correctly apply the natural laws and realize the harmonious unity between man and nature. EAE imbues educated people with a sense of beauty and encourages them to pursue and yearn for ecological beauty. Colorful ecological landscapes, endless back and forth, and endless phenomena make people feel a new rhythm of life, ideal living conditions, and a stirring sense of life, which makes them indulge and miss. Ecological beauty education will have a positive impact if we can make conscious use of these resources and educate people about ecological beauty.

The difference of EAE resources in schools is caused by the imbalance of social economy, the difference of management system and supply mode, and the information asymmetry of social demand for talents. The difference of school EAE resources constitutes the difference of school EAE process and effect. The diversity and complexity of school EAE resources determine the instability of education resources, among them are human factors, material factors, policy orientation, development and changes of social and economic conditions, and other factors. Its mobility is mainly manifested in the flow of teachers' resources and students' resources and funds.

**Figure 1:**
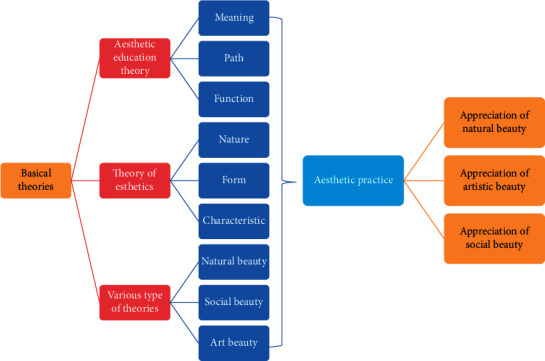
Basic framework of AE resource application in universities.

The aesthetic course of university is mainly realized through aesthetic activities, so the appreciation and creation of various kinds of beauty is an important part of the course content. If you want to appreciate various forms of beauty, you must know and understand the basic knowledge of various forms of beauty. Through the study of various meanings, features, forms, elements, and other knowledge of beauty, we can accumulate certain theoretical knowledge and become the basis of aesthetic practice. The forms of beauty can be divided into natural beauty, social beauty, and artistic beauty, and the aesthetic practice part of the application of university AE resources is also selected from these four parts. It can be concluded that the basic framework of university AE resource application is shown in Figure 1.

The frame diagram only shows what the AE courses in universities should have, which can be emphasized or supplemented in the implementation of specific courses. For example, in the part of aesthetic practice, you can appreciate and create natural beauty, artistic beauty, and social beauty at the same time, or you can choose one kind of beauty as the main part of the course and another form. Even in the part of basic theory, theoretical study can be appropriately increased or decreased according to class time, students' aesthetic quality, classroom content arrangement, and so on.

### 3.2. University AE Resources Application Optimization

#### 3.2.1. Hybrid Recommendation System

Social ecological beauty is the beauty reflected in the social ecosystem, which widely exists in all fields of people's production and life, is reflected in the harmonious and orderly social life, and embodies people's ecological aesthetic ideal. Therefore, we can cultivate people's sensibility and ecological aesthetic consciousness through various social and ecological aesthetic forms. In the implementation of EAE in schools, the development of material resources such as educational environment resources should reflect the spirit of the times, and the architectural style should fully reflect the characteristics of vitality and simplicity. At the same time, we should also pay attention to the nationalized and localized material resources, which contain our precious culture and fine traditions, and embody the harmonious unity between man and nature, tradition and modernity, and ideas and modernity.

The world, including humans, is an interconnected life ecosystem that appears as an organic whole. The concept of ecological science highlights the inseparable relationship between man and nature, breaking the existing concept of separation and opposition between man and nature. Everything in this place is connected. Life and life, life and environment, life and environment, and life and environment, communicate and coordinate with one another, cooperate with one another, displaying a beautiful form. The use of a recommendation system [24] is one of the most important and effective ways to reduce information overload, and it is crucial to the success of various Internet application platforms. Most existing recommendation algorithms are based on the CF algorithm, which analyzes historical interactive information, such as scoring, browsing, and collecting, to provide products that users are most likely to be interested in. The evaluation matrix, on the other hand, is very sparse in the face of very sparse real-world data, and the CF-based model frequently fails to provide reliable recommendations. To reduce data sparseness, cold start, and scale, the HHR_L (Learning-based Hash Hybrid Recommendation) model constructs a loss function aimed at predicting ranking and learns the effective hash codes of users and projects by adding individual constraints to users and projects.

**Figure 2:**
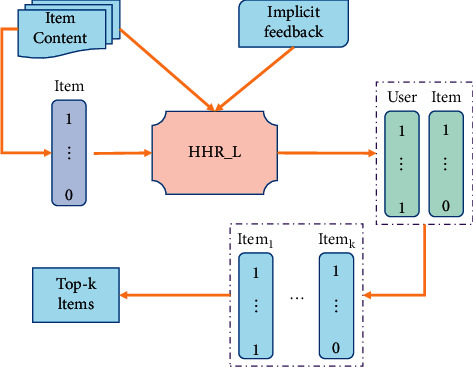
Framework diagram of HHR_L model.

The HHR_L model is proposed to solve the data sparseness, cold start, and scalability problems in recommendation system. HHR_L constructs a recommendation algorithm based on hash, which enables the recommendation system to handle large-scale data recommendation, improves the recommendation efficiency, and provides an effective method for scalable recommendation. The model framework as an example of HHR_L is shown in Figure 2.

At first, we initialize the hidden layer feature representation of the project by DAE (Denoising Autoencoder); then we fine-tune the DAE network by combining the paired sorting objective function based on CF, and finally get the hash codes and items of users, and the top-*k* suggestions are given by Hamming distance sorting.

The basic idea of hashing algorithm is to compress input data of arbitrary length and map it to data output of fixed length. A key skill is dimension reduction. For test samples, if the information from adjacent samples and related markers is known, the maximum posterior criterion is used to predict a group of markers in the test samples.

Then, through the maximum posterior probability criterion, it can be shown that the *i*th mark of sample *x* is

(1) $$\begin{array}{c} y_{x}\left(i\right)=\underset{b\in \left\{0,1\right\}}{\arg\max}P\left(H_{i}^{b}|x\right),\;i\in Y. \end{array}$$

Among them, *y*_*x*_(*i*) represents the posterior probability of the label corresponding to sample *x*. Because the posterior probability is difficult to be directly calculated, it can be converted into prior probability and conditional probability according to Bayesian theorem. Therefore, if the label of the sample is unknown, the labeled sample can be expressed as

(2) $$\begin{array}{c} y_{x}\left(i\right)=\underset{b\in \left\{0,1\right\}}{\arg\max}P\left(H_{i}^{b}\right)P\left(x|H_{i}^{b}\right), \end{array}$$

*P*(*H*_*i*_^*b*^) represents the prior probability of *H*_*i*_^*b*^, *P*(*x|H*_*i*_^*b*^) represents the conditional probability that the tag belongs to sample *x* when *H*_*i*_^*b*^ is known, and *P*(*H*_*i*_^*b*^) and *P*(*x|H*_*i*_^*b*^) can be obtained from a given data set.

And change the distribution of hashed points on the machine by selecting a specific hash function. First, given the parameter *D* > 0, the hash function *R*^*k*^⟶*Z* satisfies the formula

(3) $$\begin{array}{c} G\left(v\right)=\left[\frac{\alpha \cdot v+\beta }{D}\right], \end{array}$$

*α* ∈ *R*^*k*^ represents a *K*-dimensional feature vector that obeys *N*(0,1) standard normal distribution, *β* ∈ *R* represents a feature vector randomly selected from the range of [0, *D*], and *D* represents the width of the bucket.

From the above steps, we can get the quantization result from *k* groups *s*={*s*_1_, ⋯, *s*_*k*_}. For example, the *i*th group will have a cluster center point *μ*_*i*_. We distinguish data points based on the median points of two neighboring groups, and the median plane between them is hyperplane. The definition of the vertical plane is as follows:

(4) $$\begin{array}{c} {\left(x-\frac{\mu_{i}+\mu_{j}}{2}\right)}^{T}\left(\mu_{i}-\mu_{j}\right)=0. \end{array}$$

Given *B*, *D*, *Y*, Θ, when the intermediate variable *X* is updated, the items unrelated to *X* in formula equation (4) are eliminated, and the objective function becomes the following subobjective about *X*:

(5) $$\begin{array}{c} \underset{X\in R^{d\times n}}{\max}tr\left(B^{T}X\right),\\ \text{s.t }X1_{n}=0,\;XX^{T}=nI_{d}. \end{array}$$

The above model can be optimized by SVD (Singular Value Decomposition). In particular, the update rules of *X* are as follows:

(6) $$\begin{array}{c} X=\sqrt{n}\left[U_{b},{\hat{U}}_{b}\right]{\left[V_{b},{\hat{V}}_{b}\right]}^{T}. \end{array}$$

*U*_*b*_, *V*_*b*_ is a stack of left singular vector and right singular vector of matrix $\bar{B}$ : ${\bar{b}}_{ui}=b_{ui}-1/n\sum_{u=1}^{n}b_{ui}$ centered on row mean, respectively. ${\hat{U}}_{b}$ is a matrix of zero singular vector stack, and ${\hat{V}}_{b}$ represents Gram–Schmidt orthogonal complement of *V*_*b*_, which can be determined by ${\left[V_{b},1\right]}^{T}{\hat{V}}_{b}=0$.

#### 3.2.2. Text Classification

A collection of natural languages is referred to as a document set. To classify and organize the document set, all that is required is to extract elements from the document set that can express the content of each document. Because “word” is a fully expressible semantic object in Chinese documents, it is frequently chosen as the metadata of text features. The metadata of text features must first be extracted before text mining can begin. Currently, all types of text representation methods suffer from the same issue. There are many dimensions that can be used to represent a document, and many documents that are not useful for text clustering. As a result, text features must be extracted twice. After the text feature vectors have been dimension reduced, they can be clustered, and the clustering results can be analyzed in light of the current situation.

“Thesaurus,” or machine dictionary, has as many entries as possible. Word segmentation based on Thesaurus is the most commonly used, the simplest operation method and the most effective method. Match the item to be processed with Thesaurus, and if the match is successful, display the item string as a word. If they appear more at the same time, they are most likely to form a word. Therefore, the confidence of two adjacent words can usually be obtained by calculating their co-occurrence frequency. The mutual information *M* is

(7) $$\begin{array}{c} M\left(X,Y\right)=\log\frac{P\left(X,Y\right)}{P\left(X\right)P\left(Y\right)}, \end{array}$$

where *P*(*X*, *Y*) is the adjacent co-occurrence probability of *X*, *Y*, and *X*, *Y* represent two Chinese characters. *P*(*X*), *P*(*Y*) are the probability of *X*, *Y* appearing separately in the text. This can indicate the tightness between Chinese characters. When the value of *M* is greater than a certain threshold, it can be considered that *X*, *Y* can form a word.

CI (Conceptual index) is a simple and effective dimension reduction method. For supervised learning, CI constructs CI subspaces, takes the cyclic vector composed of these CI subspaces as the base vector, and projects the text cyclic vector onto this subspace.

Classified prototype vector: assuming that the prototype vector of the *i*th classification is Center_*i*_, its calculation method is as follows:

(8) $$\begin{array}{c} {\text{Center}}_{i}=\frac{1}{N}\overset{N_{i}}{\underset{j=1}{\sum }}{\text{Doc}}_{ij}, \end{array}$$

where *N*_*i*_ is the number of texts in category *C*_*i*_ and Doc_*ij*_ represents the *j* prototype vector in category *C*_*i*_.

PCA (principal component analysis) is a widely used method of pattern recognition and signal processing, which is especially effective for data compression and feature extraction. In this paper, we apply PCA method to text classification, hoping to obtain low-dimensional representation of text vectors.

Assuming that there are *M* categories in the training set sample, the following is how to calculate the covariance matrix of each internal document category separately:

(9) $$\begin{array}{c} X_{i}=E\left[\left(x-\mu \right){\left(x-\mu \right)}^{T}\right]. \end{array}$$

Among them, *X*_*i*_ is the covariance matrix of the internal document classification in the *i*th classification, *x* is the text vector in the *i*th classification, and *μ*_*i*_ represents the prototype vector in the *i*th classification.

The essence of PCA method is that you can always get the best linear mapping from *M*-dimensional space to *D*-dimensional space. This is because the sum of error squares of the data obtained by the algorithm can always be kept to a minimum. At the same time, the mutual information between the original vector *x* and the mapping vector *y* is the largest.

When dealing with multiclassifier combinations in classification problems, the voting mechanism is frequently used in machine learning. In the above rules, the relative majority voting rule, which selects the class with the highest output among all machine classification algorithms, is a commonly used voting rule. The RF (random forest) algorithm must aggregate the classification results of each DT in the forest and vote on classification categories when determining the classification results. The DT (decision tree) model's similarity can be calculated from two perspectives: DT semantics and DT structure. The concrete implementation idea for improving the voting mechanism is to combine the DT classification effect with the weighted voting method of class probability to improve the RF voting mechanism. The accuracy of data classification outside the bag reflects the classification effect of DT. The DT classification effect improves as classification accuracy improves.

Before making the classification decision, it is necessary to calculate the accuracy rate of each DT's out-of-bag data. If the total number of samples is *X* and the number of correctly classified samples is *X*^correct^, then the accuracy rate of DT's out-of-bag data is the ratio of the two. The accuracy of DT data outside the bag is used to weight DT, and the weight of DT in RF algorithm is

(10) $$\begin{array}{c} W_{i}=\frac{X^{\text{correct}}\left(i\right)}{X}. \end{array}$$

Remember that the probability that DT outputs that the sample belongs to each class is *P*_*i*_, the probability that the sample belongs to class *y* is *P*_*i*_(*y*), the weight of DT is *w*_*i*_, and the number of DTs in RF is *k*. The weighted value *z*(*y*) of the sample belonging to class *y* is obtained by the formula

(11) $$\begin{array}{c} z\left(y\right)=\sum_{i=1}^{k}w_{i}*P_{i}\left(y\right). \end{array}$$

The probability that the sample belongs to each class is calculated by the above method, and the class with the largest weighted value is selected as the classification result.

**Figure 3:**
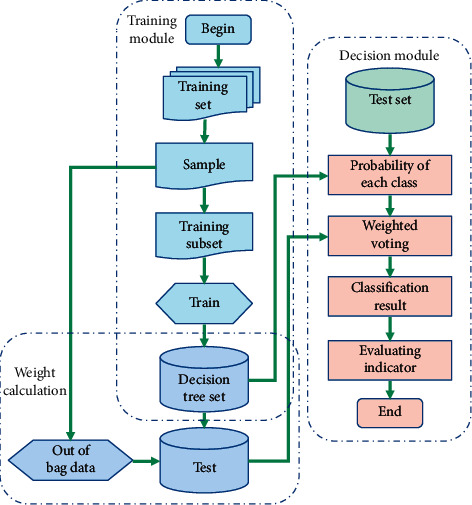
RF algorithm for optimizing voting mechanism.

The RF_VM (Random Forest Classification Using Optimized Voting Mechanism) model can be divided into three modules: learning module, weight calculation module, and decision module. The algorithm flow is shown in Figure 3.

First, we implement a training module, which is almost the same as the traditional RF algorithm. Next, the weight calculation module calculates the weight of each DT according to formula (10). Finally, the decision-making module uses the DT set obtained by the learning module and the DT weight obtained by the weight calculation module to classify the samples and calculates the probability weight that the samples belong to a specific category as formula (11). Compare the probabilities of all classes, and get the classification results by weighted values.

## 4. Experiment and Results

### 4.1. Experimental Environment Setting

The computer used in the experiment is running Microsoft Windows 10. In this article, we choose Python as the development language of our experiment. Try PyCharm software to make Python language development more efficient.

As an excellent Python integrated development software, PyCharm can provide intelligent Python support through intelligent code completion, code inspection, and navigation. PyCharm supports project and file management and various scientific packages, such as Anaconda, matplotlib, and NumPy.  Table 1 shows the main hardware configuration of the computer used in this experiment.

In order to verify the effectiveness of the proposed algorithm in cold start and sparse scenarios, we choose to experiment on two public datasets (Amazon dataset and Yelp 9th round dataset), and the sparsity is as high as 99.9%. For content data, first, delete punctuation marks and numbers. And words with stop words and length less than 2 usually have no clear meaning, so the remaining words are processed by English word segmentation.

### 4.2. Result Analysis

**Figure 4:**
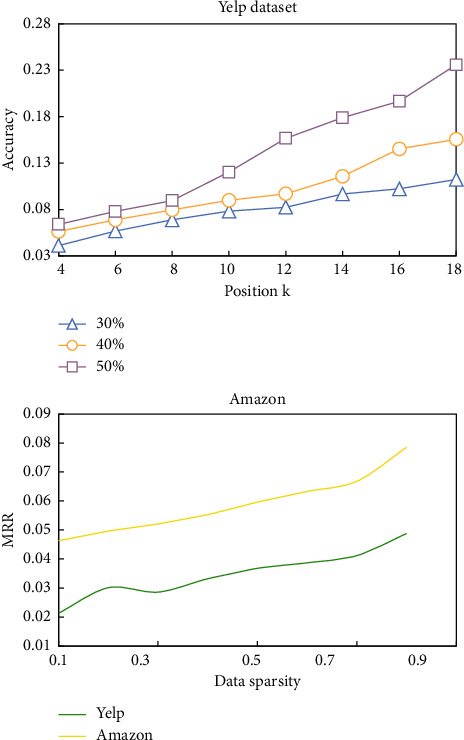
The recommendation accuracy of HHR_L in sparse environment varies with data sparsity.

In this paper, Accuracy and MRR (Mean Reciprocal Rank) are used to evaluate the recommendation performance of HHR_L in cold start environment. The experimental results are shown in Table 2 and Figure 4.

It can be seen that the recommendation accuracy of HHR_L is higher than that of comparison method. This is because [18] and [20] are hash recommendation algorithms based on CF, and no project content data is used for modeling. However, in a highly sparse environment, the recommendation accuracy of [18] and [20] will be reduced because the content data plays a key role in improving the recommendation accuracy.

In addition, although the recommendation accuracy of HHR_L increases steadily with the increase of data density, the performance of [21] is unstable, because [21] is based on score prediction rather than ranking. Because the prediction goal is consistent with the ultimate goal of the recommendation system, the recommendation performance is more reliable.

**Figure 5:**
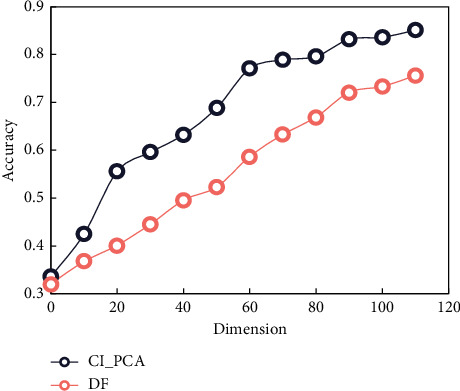
Comparison of dimension reduction effects.

Keep more commonly used words in text documents to improve classification accuracy. CI_PCA and DF (Document frequency) are compared in dimension reduction effect, so KNN (*K*-Nearest Neighbors) algorithm is used for classification, where *K*=10 is shown in Figure 5.

In Figure 5, except for the 0 dimension, we can see that the accuracy of CI_PCA proposed in this paper is much higher than that of the conventional DF method. When the last 110 dimensions are reached, the accuracy of the algorithm is as high as 80%, and the accuracy of DF method is only 70%. Increasing the dimension to 5 significantly improves the accuracy of the text frequency method.

**Figure 6:**
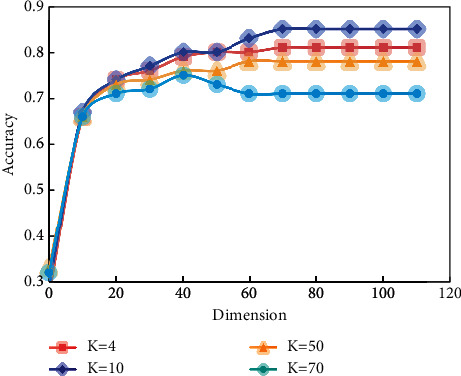
Influence of *K* value on accuracy.

Another problem in this example is how to choose the appropriate *K* value when using KNN algorithm for classification. The choice of *K* value has a great influence on the classification effect. If the *K* value is too small, the similarity vector may be ignored. If the *K* value is too large, a large number of noise vectors will seriously affect the classification result. The results are shown in Figure 6.

The experimental results show that the small *K* value is better than the large *K* value, and the accuracy rate can reach 81% when *K*=10 is 110. That is to say, after the CI_PCA process, we prove that similar documents are closer in subspace, prototype vector can weaken the negative influence of synonyms and semantics, and projection of original text vector can partially filter “document vector.”

**Figure 7:**
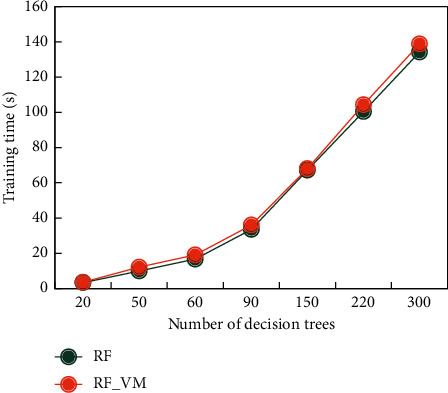
Algorithm time comparison.

In this paper, the existing RF classification algorithm and RF_VM algorithm are used for the comparative experiment of text classification. Different DT numbers are selected for text classification. In the experiment, the number of DT trees is selected as 20, 50, 60, 90, 150, 220, and 300, and other hyperparameters are used as default values for comparative experiments. The time for performing text classification is shown in Figure 7.

**Figure 8:**
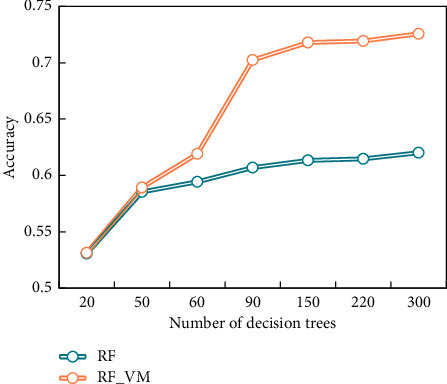
Comparison of prediction accuracy of algorithms.

It can be seen from Figure 7 that the number of DTs in RF classification algorithm has a certain influence on the text classification time of both algorithms. Most of the text classifications using these two algorithms will increase accordingly. Although the RF_VM algorithm in this paper is more complicated than the existing RF algorithm in voting, each DT is given a constant weight according to the prediction accuracy of the trained out-of-pocket data. However, the probability that the output samples belong to each category does not significantly increase in the required time resources.  Figure 8 shows the prediction accuracy of the existing RF classification algorithm and the RF_VM algorithm in this paper for text classification experiments.

It can be seen that the number of DTs in the algorithm has a great influence on the accuracy of the algorithm. Generally speaking, the higher the number of DTs, the higher the prediction accuracy of the algorithm. For other DT trees, the prediction accuracy of RF_VM algorithm in this paper is improved compared with the existing RF algorithm. The RF_VM algorithm in this paper improves the prediction accuracy of text classification based on the characteristics of less increase of time resources and better performance than existing RF algorithms. In addition, the prediction accuracy of the two algorithms failed to reach a high level, which was related to the use of default values (excluding DT number) for the superparameters of the algorithms, which also proved the importance of value optimization.

## 5. Conclusions

EAE is extremely important for environmental protection and improving human living conditions. The EAE educational method should focus on aesthetics, psychological discipline, and infection, and its practical implementation should be integrated with all aspects of school operations. Teachers' human resources are the main body, curriculum resources are the foundation, and material resources are the carrier in the development of school EAE resources, in order to create a humanized environment, build an information platform, and carry out various activities. When designing an ML algorithm, try to avoid data sparsity and a cold start. Data scarcity is not a big deal because of the curriculum limitations, so we pay attention to the new curriculum. By projecting the high-dimensional prototype text onto a low-dimensional subspace and then reducing the dimension according to the classification covariance matrix, the CI PCA algorithm significantly reduces the classification cost. When the proposed RF algorithm is combined with the optimized voting mechanism, the algorithm's classification ability can be greatly improved.

## Figures and Tables

**Table 1:** Use the main hardware configuration of the experimental computer.

Project	Deploy
CPU	Intel Core i5 2.5 GHz
Internal storage capacity	8 GB
Hard disc capacity	300 GB

**Table 2:** Comparison of recommended accuracy at sparsity of 20% and 30%, respectively.

Method	Amazon		Yelp	
	20%	30%	20%	30%
Ref [18]	0.0123	0.0169	0.0196	0.0341
Ref [20]	0.0114	0.0102	0.0192	0.1162
Ref [21]	0.0463	0.0266	0.0154	0.0128
HHR_L	0.0452	0.0511	0.0288	0.0286

## Data Availability

The data used to support the findings of this study are included within the article.
